# Contribution of medico-administrative data to the development of a comorbidity score to predict mortality in End-Stage Renal Disease patients

**DOI:** 10.1038/s41598-020-65612-x

**Published:** 2020-05-22

**Authors:** Adélaïde Pladys, Cécile Vigneau, Maxime Raffray, Bénédicte Sautenet, Stéphanie Gentile, Cécile Couchoud, Sahar Bayat

**Affiliations:** 1 Univ Rennes, EHESP, REPERES (Recherche en pharmaco-épidémiologie et recours aux soins) – EA 7449, F-35000 Rennes, France; 2 University of Rennes 1, INSERM, U1085-IRSET Rennes, France; 3 CHU Pontchaillou, Department of Nephrology, Rennes, France; 4 Tours Hospital, Department of Nephrology-Hypertension, Dialysis, Kidney Transplantation, Tours University, INSERM UMR, 1246 SPHERE Tours, France; 5 University of Aix-Marseille, CEReSS (Centre d’Études et de Recherche sur les Services de Santé et la Qualité de Vie) - EA 3279, Marseille, France; 6 Renal Epidemiology and Information Network (REIN), Biomedecine Agency, Saint Denis La Plaine, France

**Keywords:** Nephrology, Risk factors

## Abstract

Comorbidity scores to predict mortality are very useful to facilitate decision-making for personalized patient management. This study aim was to assess the contribution of medico-administrative data in addition to French Renal Epidemiology and Information Network (REIN) data to the development of a risk score to predict the 1-year all-cause mortality in patients with End Stage Renal Disease (ESRD), and to compare it with previous scores. Data from a derivation sample (n = 6336 patients who started dialysis in 2015 in France) obtained by linking the REIN and the French National Health Insurance Information System databases were analyzed with multivariate Cox models to select risk factors to establish the score. A randomly chosen validation sample (n = 2716 patients who started dialysis in 2015) was used to validate the score and to compare it with the comorbidity indexes developed by Wright and Charlson. The ability to predict one-year mortality of the score constructed using REIN data linked to the medico-administrative database was not higher than that of the score constructed using only REIN data (i.e., *Rennes score*). The Rennes score included five comorbidities, albumin, and age. This score (AUC = 0.794, 95%CI: 0.768–0.821) outperformed both the Wright (AUC = 0.631, 95%CI: 0.621–0.639; p < 0.001) and Charlson (AUC = 0.703, 95%CI: 0.689–0.716; p < 0.001) indexes. Data from the REIN registry alone, collected at dialysis start, are sufficient to develop a risk score that can predict the one-year mortality in patients with ESRD. This simple score might help identifying high risk patients and proposing the most adapted care.

## Introduction

The number of patients with End Stage Renal Disease (ESRD) continues to increase in France and worldwide, particularly due to aging and the increased prevalence of type 2 diabetes^1^. Besides these risk factors, patients with ESRD often present several comorbidities (cardiovascular disease, hypertension) associated with poor survival. Moreover, dialysis start conditions could be associated with lower survival^2^. However, good tools to predict mortality are lacking. Therefore, it could be useful to develop new tools to identify high risk patients for proposing the most adapted care.

To assess survival in patients with ESRD, comorbidities should be taken into account. A comorbidity score allows summarizing several risk factors to predict outcomes (e.g., mortality, hospital stays) and also to describe the comorbidity burden of specific populations. The Charlson comorbidity index^3^ is the most widely used score, in the clinic and in studies, to assess survival also of patients with ESRD^4–7^. It was developed in patient populations with various diseases, and it is not disease-specific. Several authors tried to develop comorbidity scores to specifically predict outcomes in patients with ESRD or on dialysis^8–11^. LF Wright was the first to develop a comorbidity score specific for patients starting dialysis^8^. Afterwards, only few authors developed new comorbidity scores adapted to specific ESRD populations, such as patients on hemodialysis^9^, elderly patients with ESRD^11–13^, and patients with ESRD covered by Medicare^10,14^.

With the increasing use of medico-administrative databases in healthcare, some authors have developed scores that exploit administrative data^15–18^. Deyo *et al*., were the firsts to adapt the Charlson comorbidity index to administrative data of patients hospitalized for lumbar spine surgery, by identifying the ICD Ninth Edition (ICD-9) codes^15^. In France, Bannay *et al*., adapted the Charlson comorbidity index to the French National Health Insurance database (SNDS), using ICD-10 codes^16^. Liu *et al*., developed a new comorbidity score using ICD-10 codes from the Medicare ESRD database^10^.

The literature on the use of comorbidity scores is extensive, including for predicting the mortality of patients with ESRD or on dialysis^14^. Nevertheless, previous risk scores to predict mortality of patients with ESRD could not be generalized because they were developed using specific populations^9–14,19^, or a long time ago (20–30 years)^8^. Consequently, many authors preferred to use the Charlson comorbidity index^4–7^. Nevertheless, two works recently showed that the existing comorbidity scores, including the Charlson index, do not accurately predict mortality in patients with chronic kidney disease^20^ and on dialysis^21^.

In France, the Renal and Epidemiology Information Network (REIN) database^11–13^ has been used to develop scores to predict the 6-month prognosis^11^ and to improve the patient-centered care and decision-making of elderly patients with ESRD at dialysis start^22^. Nevertheless, no study focused on the development of a new comorbidity score to predict the 1-year mortality of all French patients at dialysis start. In addition, no study assessed the contribution of medico-administrative data to the establishment of a comorbidity index to predict the one-year survival of patients with ESRD.

The aim of this study was to (i) develop a simple, useful risk score, not depending on dialysis parameters, to predict the 1-year all-cause mortality of patients with ESRD, using REIN data and also data obtained by linking the REIN and SNDS databases; (ii) compare the predictive performance of this score and of previous comorbidity indexes; and (iii) compare the predictive performance of this score in patients with emergency first dialysis and patients with planned first dialysis.

## Methods

### Study population

All incident ≥18-year-old patients from REIN^23^ who started dialysis (hemodialysis or peritoneal dialysis) in France in 2015 were included. Patients were randomly separated in two subgroups with a 70:30 proportion: derivation sample and validation sample.

### Database linkage procedure

France has an extensive medical and administrative information system: French national health insurance information system (SNDS). This system covers about 96% of the inhabitants living in France and all their health care expenditure reimbursement by national health insurance. Consequently, the SNDS contains individual, anonymous, and comprehensive data on all health expenditure reimbursements for patients. In addition, the SNDS contains discharge diagnoses (with ICD-10 codes) and medical procedures performed during each hospital stay. Nevertheless, diagnoses performed during general practitioner or physiotherapy consultations cannot be detected in the SNDS.

To complete the patients’ baseline (at dialysis start) characteristics, data from the REIN registry were linked to data from the SNDS. As both databases contain anonymized information, a deterministic linkage method was developed to merge information from the two databases based on: sex, age, month of dialysis start, center of first dialysis, and postcode of residence.

### Collected data

Data from the SNDS database were used to identify the comorbid diseases (ICD-10 codes) included in the Charlson index^3^ from hospital stays up to 2 years before dialysis initiation (Supplementary material, Table S1). Data collected from the REIN registry at dialysis start were age and albumin levels; comorbidities including cardiovascular diseases (coronary artery disease, peripheral vascular disease, congestive heart failure, arrhythmia, aneurism and cerebro vasculardisease), active malignancy (all solid tumors and hematological malignancies), hepatic disease, diabetes, respiratory insufficiency (all pulmonary disease requiring a treatment or causing several hospitalizations), and walking disability (walks without help, needs partial assistance for transfers, totally dependent for transfers). Derivation and validation sample characteristics were compared in Supplementary Table S2. Comorbidity-related data in REIN could be completed with the diagnoses associated with the hospital stays before dialysis initiation from the SNDS database (Table 1). Date and causes of death were collected from the REIN registry.

**Table 1:** Risk factors used to establish the comorbidity scores.

Factors	Factors sources	Scores		
		Rennes	Charlson	Wright
Age	R, S	✓	✓	✓
Albumin level	R	✓		
Cardiac diseases^a^	R, S	✓		✓
Myocardial infarction	R, S		✓	
Congestive heart failure	R, S		✓	
Peripheral vascular disease	R, S		✓	
Cerebrovascular disease	R, S		✓	
Aortic aneurism	R			
Arrhythmia	R			
Coronary artery disease	R			
Dementia	S		✓	
Respiratory insufficiency	R, S	✓	✓	✓
Connective tissue disorder	S		✓	
Peptic ulcer disease	S		✓	
Hepatic disease *(unspecified)*	R,S	✓		
Mild liver disease	S		✓	
Moderate or severe liver disease	S		✓	
Diabetes *(unspecified)*	R,S			✓
Diabetes without chronic complication	S		✓	
Diabetes with chronic complication	S		✓	
Hemiplegia or paraplegia	R, S		✓	
Active malignancy^b^	R,S	✓		✓
Any malignancy (no metastatic)^c^	S		✓	
Metastatic solid tumor	S		✓	
Walking disability	R	✓		

^a^All factors were tested in the univariate Cox model, but only factors with a p-value <0.2 were included in the multivariate model; ^a^In REIN, cardiac diseases included coronary artery disease, myocardial infarction, peripheral vascular disease, congestive heart failure, arrhythmia, aortic aneurism, and cerebrovascular disease; ^b^In REIN, cancer included all solid tumors and hematological malignancies; ^c^Any malignancy = lymphoma, leukemia and all solid tumors; R: REIN; S: SNDS; ✓: risk factor included in the score.

This study is approved by the French data protection authority (Commission nationale de l’informatique et des libertés – CNIL –; agreement number: 917021) and by the scientific committee of the French Biomedecine Agency. The CNIL is the institution in France who delivers granted authorization to use data for a study. Verbal informed consent to participate was obtained from all subjects involved. For this study, all research was performed in accordance with relevant guidelines.

### Scores

#### Rennes comorbidity score establishment and validation

The Rennes score was computed using the derivation sample. All variables collected in the REIN and SNDS databases were first tested in univariate Cox models. All variables with a p-value <0.20 in univariate models were included in the multivariable Cox model. On the basis of the univariate model results, a first score was developed using REIN and SNDS data (model 1), using a Cox model to assess the influence of each comorbid disease on the 1-year mortality (hazard ratio, HR). Then, a second score was established using only REIN data (model 2). Finally, all HR values estimated in the derivation sample and significantly associated (p < 0.05) with 1-year mortality in the multivariate model were converted into index weights as follow: an HR of 1.2 to 1.5 received a weight of 1, an HR of 1.5 to 2.5 received a weight of 2, an HR of 2.5 to 3.5 received a weight of 3, and so forth. In addition, 1 point for each decade of age after the age of 50 years was added to the total score. The comorbidity score for each patient was the sum of the weights based on the presence or absence of each condition.

Then, the weights estimated in the previous step were assigned to patients in the validation sample. The discriminatory ability of the predictive risk-score model was assessed using the area under the receiver operating characteristic (ROC) curve (AUC). In our study, the AUC quantified the ability of our scores to assign a high probability of death to patients who died. Values ranged from 0.50 (no ability to discriminate) to 1.0 (perfect discrimination). All scores were compared and cross-checked to identify the score with the best ability to predict the 1-year mortality. The calibration curve evaluates the accuracy in different subgroups at risk.

Before the implementation of the survival models in the derivation sample, missing data were handled by using multiple imputation by chained equations (MICE) with ten imputations and five cycles^24^. The score validation was performed using the complete dataset in the validation sample.

#### Comparison with the Charlson and Wright comorbidity indexes

To assess the ability of our new score to predict 1-year all-cause mortality, two previous comorbidity indexes were used: i) the original and the age-adjusted Charlson comorbidity indexes, and ii) the Wright comorbidity index. As the REIN registry did not include all the comorbidities used to establish the Charlson score, the Charlson index was constructed using data charts from the SNDS database. The Wright comorbidity index is a combination of age and comorbid conditions, leading to three risk groups: low, medium, and high risk^8^. ROC curves were constructed and the discriminatory abilities of the three scores were compared using the AUC, based on each regression model predictions.

### Software

The linkage procedure was established with SAS and R. Scores and analyses were performed with the STATA 13.1 software. The application was developed using the R-shiny package with R.

### Ethical approval

Subjects involved in our study were extracted from the French REIN registry which received the agreement from the CNIL (Commission Nationale de l’Information et des Libertés) in 2010 (agreement number: 903188 Version 3). Verbal informed consent to participate was obtained from all subjects involved. This study is approved by the CNIL (agreement number: 917021) and by the scientific committee of the French Biomedecine Agency.

## Results

### Study population

**Figure 1:**
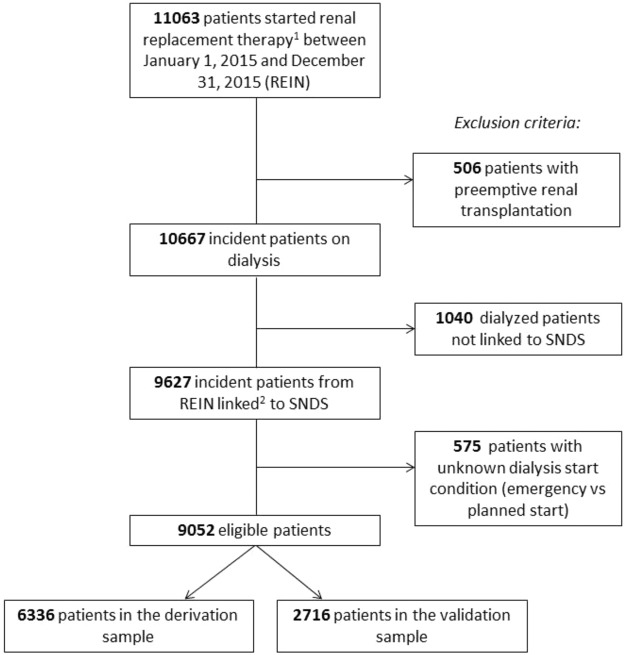
Flowchart of the patients’ inclusion procedure. ^1^Renal replacement therapy includes: preemptive renal transplantation or dialysis (peritoneal dialysis or hemodialysis); ^2^Data from the REIN and SNDS databases were merged on: sex, age, month of dialysis start, center of first dialysis and postcode of residence.

Through the linkage procedure, 90.3% of REIN patients (n = 9627) could be identified in the SNDS database. A total of 9052 incident patients on dialysis were included in our study (Fig. 1), with a sex (M/F) ratio of 1.81 and a mean age at dialysis start of 68.4 ± 15.1 years (Supplementary material Table S2). During the first year of follow-up, 1302 (14.4%) patients died. Causes of death were presented in the Supplementary material (Table S3).

### Rennes score establishment and validation

#### Score establishment

Our score was established using the derivation sample (n = 6336 patients) and the variables described in Table 1. These variables were extracted from the REIN and were completed/corrected using data from the SNDS database. This lead to modifying the diabetes status (1 patient with missing diabetes status and 103 diabetes-free patients in the REIN registry were diabetics; 1.15%), the malignancy status (6.05%), respiratory insufficiency status (5.1%), and hepatic disease status (3.3%) in several patients. First, all variables were tested with univariate Cox models (see Supplementary Table S2 Table S4). Two simplified models were established using multivariate models: i) model 1 included data from the REIN and SNDS databases (Table 2, left panel), and ii) model 2 included only data from the REIN database (Table 2, right panel).

**Table 2:** Risk factors for 1-year all-cause mortality prediction in multivariate Cox models, using data from the REIN and SNDS databases (model 1), or REIN alone (model 2) and the associated weights in the derivation sample (n = 6336).

	Model 1		Model 2 (*Rennes score*)	
	Adjusted HR (95% CI)	Weights	Adjusted HR (95% CI)	Weights
Age (years)	1.04 (1.03–1.05)	1	1.04 (1.03–1.04)	1
**Albumin** ***(vs*** ≥ ***30*** ***g/dl)***				
<30	1.83 (1.58–2.11)	2	1.82 (1.57–2.10)	2
**Active malignancy** ***(vs No)***				
Yes	n/a	n/a	2.25 (1.95–2.59)	2
Any tumor *(vs No)*				
Yes	1.96 (1.65–2.33)	2	n/a	n/a
**Metastatic solid tumor** ***(vs No)***				
Yes	3.64 (2.64–5.03)	4	n/a	n/a
**Hepatic disease** ***(vs No)***				
Yes	n/a	n/a	1.44 (1.15–1.80)	1
**Respiratory insufficiency (vs No)**				
Yes	1.30 (1.12–1.51)	1	1.30 (1.12–1.52)	1
Moderate to severe liver disease *(vs No)*				
Yes	2.25 (1.55–3.25)	2	n/a	n/a
**Walking disability** ***(vs Autonomy)***				
Moderate	2.26 (1.90–2.69)	2	2.24 (1.89–2.66)	2
Severe	3.73 (3.06–4.55)	4	3.61 (2.96–4.41)	4
**Cardiovascular diseases** ***(vs 0)***				
1	1.68 (1.40–2.02)	2	1.66 (1.38–1.98)	2
≥2	2.21 (1.87–2.61)	2	2.19 (1.85–2.58)	2

#### Score validation

Scores (from model 1 and model 2) were validated in the validation sample (n = 2716 patients). The weights calculated in the previous step were attributed to the validation population. Scores from model 1 varied between 0 and 16, and scores from model 2 varied between 0 and 14. Both models showed a similar ability to predict the one-year all-cause mortality (model 1: AUC = 0.789; 95%CI: 0.761–0.816; and model 2: AUC = 0.794; 95%CI: 0.768–0.821 p = 0.239) (Table 3). Therefore, model 2 (only REIN data) was chosen as risk score to predict the 1-year mortality and was called the *Rennes score*. For the Rennes score establishment, the following items were selected: age (1 point for each decade ≥50 years), albumin <30 g/dl (2 points), active malignancy (2 points), hepatic disease (1 point), respiratory insufficiency (1 point), walking disability (moderate: 2 points; severe: 4 points), and ≥1 cardiovascular disease (2 points).

**Table 3:** Univariate Cox model for 1-year mortality prediction and performance of each model in the validation population (n = 2716).

	Univariate Cox HR (95% CI)	AUC* (95%CI)
**Wright comorbidity index**		
Qualitative *(vs moderate)*		0.631 (0.621–0.639)
Low	0.45 (0.23–0.87)	
High	3.23 (2.20–4.73)	
**Original Charlson comorbidity index**		
Continuous	1.18 (1.14–1.23)	0.622 (0.606–0.638)
Qualitative *(vs 0)*		0.621 (0.605–0.636)
1	1.38 (1.01–1.88)	
2	2.27 (1.71–3.02)	
3	1.96 (1.37–2.81)	
4	2.32 (1.55–3.47)	
≥5	3.20 (2.33–4.40)	
Age-adjusted Charlson comorbidity index Continuous	1.23 (1.19–1.27)	0.703 (0.689–0.716)
Qualitative *(vs[0–2])*		0.692 (0.678–0.705)
^3,4^	2.63 (1.44–4.79)	
^5,6^	6.34 (3.59–11.18)	
≥7	10.16 (5.78–17.87)	
Score from model 1		
Continuous	1.39 (1.34–1.44)	0.789 (0.761–0.816)
**Rennes score (model 2)**		
Continuous	1.45 (1.39–1.52)	0.794 (0.768–0.821)
Qualitative *(vs[0–3])*		0.775 (0.748–0.802)
^4–6^	4.70 (2.26–9.80)	
^7–9^	13.71 (6.70–28.06)	
^10–12^	44.23 (21.44–91.21)	

HR: Hazard Ratio; CI: Confidence Interval; *AUC was calculated for each model (continuous or categorical score).

**Figure 2:**
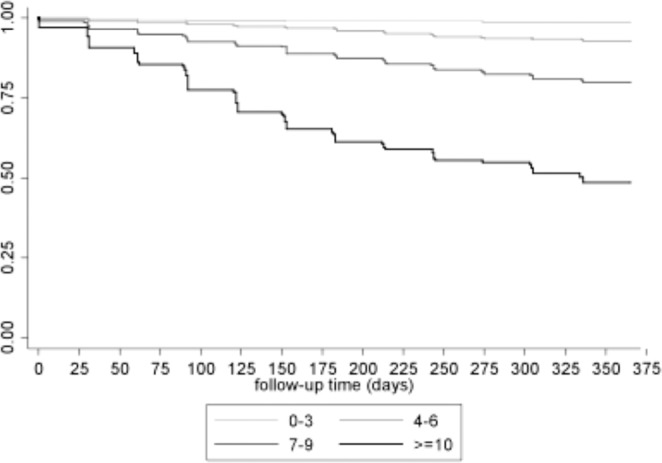
Kaplan Meier survival curves in patient subgroups according to the Rennes score (model 2): 0–3, 4–6, 7–9, and 10–12.

The median Rennes score was 6 (IQR: 4–7). Based on the Rennes score, patients in the validation sample were classified in four subgroups: ≤3 (23.2%), 4–6 (41%), 7–9 (27.5%), and 10–12 (8.2%). The mortality rates ranged from 1.6% in the lowest risk group (score ≤ 3) to 51.5% in the highest risk group (score 10–12) (Fig. 2). The probability of death increased with the score (HR = 1.45; 95%CI: 1.39–1.52) (Table 3), indicating good calibration. In agreement, the calibration curve showed a strong linear relationship between the predicted and observed 1-year mortality (R^2^ = 0.984) (see Supplementary Material Fig. S1).

### Comparison with previous comorbidity indexes

Then, the Rennes score was compared to two previous comorbidity indexes using the validation population. According to Wright’s classification^8^, 16% patients were in the low group, 17% in the intermediate risk group, and 67% in the high risk group. The predictive ability of the Wright classification was lower (AUC = 0.631; 95%CI: 0.621–0.639) than that of the Rennes score (Table 3).

Comparison with the original and age-adjusted Charlson comorbidity indexes showed that the predictive ability of the score increased when age was included (AUC = 0.622; 95% CI: 0.606–0.638 and AUC = 0.703; 95% CI: 0.689–0.716, respectively), but remained lower than that of the Rennes score (p < 0.001). In conclusion, the Rennes score ability to predict the one-year mortality was higher than that of the Wright and Charlson comorbidity indexes.

**Figure 3:**
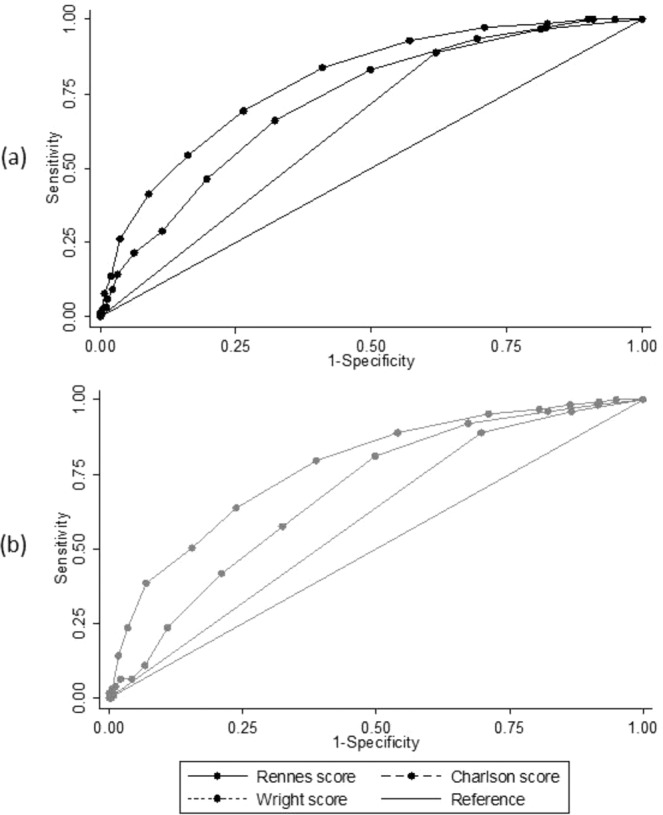
ROC curves of the Rennes score, age-adjusted Charlson comorbidity index, and Wright comorbidity index established in two subgroups of the validation sample: patients with planned first dialysis (**a**) and patients with emergency first dialysis (**b**).

Then, the predictive performance of all three scores was compared in patients (validation sample) classified according to the first dialysis conditions (emergency start vs planned start; Fig. 3(a),(b) and Table 4). The Rennes score ability to predict the one-year mortality was slightly higher (not significant) in the group with planned first dialysis (AUC = 0.794; 95%CI: 0.759–0.828) compared with emergency start (AUC = 0.777; 95%CI: 0.733–0.821).

**Table 4:** Area under the curve for patients grouped according to the dialysis start condition in the validation sample (n = 2716).

Comorbidity score	Planned start AUC (95%CI)	Emergency start AUC (95%CI)
Rennes	0.794 (0.759–0.828)	0.777 (0.733–0.821)
Charlson + age	0.725 (0.686–0.763)	0.688 (0.64–0.735)
Wright	0.639 (0.612–0.666)	0.598 (0.564–0.631)

## Discussion

In this study, we described the development and validation of a simple comorbidity score that summarizes in one index several risk factors to predict the one-year mortality in patients with ESRD. Our results suggest that the inclusion of comorbidities recorded at dialysis start in the REIN database, and not depending on dialysis parameters, is sufficient to construct a score to predict the one-year mortality risk. Indeed, additional information from the medico-administrative database did not increase the score ability to predict mortality. Nevertheless, diagnoses derived from hospital stays in the two years before dialysis initiation (extracted from the SNDS database) allowed us to verify and complete REIN data. Moreover, the new Rennes score is a good predictor of mortality and outperformed previous scores (Charlson and Wright comorbidity indexes). This score let clinicians to identify patients having a high risk of one-year-mortality before dialysis initiation and could help them to improve the patients’ personalized management regarding to dialysis initiation.

Our new score is simple to use because it has been established using only five comorbidities, one laboratory parameter, and age at dialysis start. This score could be calculated even before dialysis start because no dialysis-dependent item was retained. We observed that the first dialysis condition (in emergency or as a planned procedure) did not significantly modify the Rennes score.

Despite the use of only seven variables, the Rennes score outperformed recent scores (not directly compared in this study) developed using a large European cohort of patients on hemodialysis^9^ and data from the United States Renal Data System^10^. Floege’s score included many factors, but the observed AUC (0.73) was “acceptable, but not excellent”^9^. This score is not easy to use because it requires collecting several biological parameters (e.g., ferritin, LDL-cholesterol…). Moreover, it is not generalizable to all patients with ESRD because it was constructed specifically for people on hemodialysis. Liu *et al*., established a score based on 11 comorbid conditions in addition to the primary renal disease. Liu’s score outperforms Charlson comorbidity index, but its ability to predict mortality is low (AUC = 0.669). Moreover, it was developed using data from patients dialyzed in the early 2000s^10^, and patients’ medical conditions at dialysis start and dialysis practices might have changed in the last years.

In our study, we compared our score with the Wright comorbidity index, developed in the early 1990s and adapted specifically to a small population of patients with ESRD^8^, and also to the well-known Charlson comorbidity index^3^, developed in the general population in the 1980s. The Wright comorbidity index, as defined by the author, did not allow categorizing our population in three homogeneous subgroups and could not predict mortality (AUC = 0.631), as recently observed by McArthur *et al*.^20^. Wright’s index was developed using data from a small population of patients with ESRD dialyzed in the same unit between 1984 and 1988. Moreover, it was based on literature data of that time suggesting that early survival on dialysis was limited mainly by age and presence of diabetes or coronary artery disease. Nevertheless, in our study, diabetes was not significantly associated with the risk of death, and this variable was not included in the Rennes score. This result could be explained by the fact that diabetes treatment has changed in these last decades and this condition is currently not considered as a major risk of death for dialyzed patients. Consequently, due to the changes in the management of dialyzed patients, the Wright comorbidity index cannot adequately predict the survival of patients with ESRD and should be updated.

We then compared our score to the Charlson comorbidity indexes. First, we used the score that included 15 comorbidities (leukemia, lymphoma and solid tumors were grouped in one variable, and none of our patients had HIV/AIDS). Renal disease was not considered in the score because all dialyzed patients had ESRD. In our cohort, the original Charlson comorbidity index had a low ability to predict the 1-year mortality (AUC = 0.622). This improved when the patient’s age was included in the score (AUC = 0.703). Indeed, without the age variable, a large percentage of elderly patients with few comorbid conditions were grouped in the low-risk group. After the inclusion of age, the score distribution was more parsimonious and elderly patients were included in the higher-risk group. These results confirmed the value of age in a comorbidity score and its association with survival as previously observed^9,25^.

Inclusion of data from hospital stays that occurred two years before dialysis initiation (SNDS database) did not improve the prediction ability of our score compared with the model based only on risk factors from the REIN database. This indicates that data from the REIN registry are sufficient to develop a strong score; however, data from the SNDS allowed completing missing data because comorbidities are not mandatory items in the REIN registry. For instance, if a patient was hospitalized for a cancer two years before dialysis initiation, but the item was missing or filled as absence of cancer in the REIN registry, we could modify the cancer status of this patient. Our approach was to complete data from the REIN database using diagnoses from the SNDS database, but not to assess the quality of the registry, as performed earlier in Canada^26,27^, Australia and New Zealand^28^ and also in the United States^29^. Indeed in our study, 33.2% of included patients did not have any hospital stay during the two years before dialysis initiation, and therefore this complementary analysis to complete/confirm their comorbidity list could not be done for all patients.

The strengths of our study are that we established a simple mortality risk score based on few variables that are easy to collect. We developed an open access application in English and French to easily calculate the Rennes score (https://apladys.shinyapps.io/Rennes_score/). We tested and cross-checked two models to identify the contribution of a medico-administrative database to the establishment of our comorbidity score. Finally, we used only data from the REIN registry to develop the Rennes score that displays a good ability to predict the one-year mortality in dialyzed patients. Moreover, we showed that the Rennes score outperforms the widely used Charlson comorbidity index and also the Wright score developed for dialyzed patients. In addition, thanks to a linkage procedure established by our team, we could link SNDS data to the REIN registry for the first time in France.

Our study has also several limitations. If patients were not hospitalized during the two years before dialysis initiation, their Charlson score could not be calculated. Consequently, we might not have the full comorbidity picture of all our patients. Moreover, we validated our scores using incident French patients, whereas an external validation population could have been more suitable.

## Supplementary information

Supplementary material.
