# Supplementary material for: Contribution of medico-administrative data to the development of a comorbidity score to predict mortality in End-Stage Renal Disease patients
Source: Sci Rep. 2020 May 22;10:8582. doi: 10.1038/s41598-020-65612-x (PMC7244576; doi:10.1038/s41598-020-65612-x)
Supplement: Supplementary file 1 — Supplementary material. [file 41598_2020_65612_MOESM1_ESM.docx]

**Contribution of medico-administrative data to the development of a comorbidity score to predict mortality in End-Stage Renal Disease patients**

Adélaïde Pladys^1*^, Cécile Vigneau^2-3^, Maxime Raffray^1^, Bénédicte Sautenet^4^, Stéphanie Gentile^5^, Cécile Couchoud^6^, Sahar Bayat^1^

^1^Univ Rennes, EHESP, REPERES (Recherche en pharmaco-épidémiologie et recours aux soins) – EA 7449, F-35000 Rennes, France; ^2^University of Rennes 1, INSERM U1085-IRSET, Rennes, France; ^3^CHU Pontchaillou, Department of Nephrology, Rennes, France; ^4^Tours Hospital, Department of Nephrology-Hypertension, Dialysis, Kidney Transplantation, Tours University, INSERM UMR 1246 SPHERE, Tours, France; ^5^University of Aix-Marseille, CEReSS (Centre d’Études et de Recherche sur les Services de Santé et la Qualité de Vie) - EA 3279, Marseille, France; ^6^Renal Epidemiology and Information Network (REIN), Biomedecine Agency, Saint Denis La Plaine, France

*corresponding author: Adélaïde Pladys ([adelaide.pladys@ehesp.fr](mailto:adelaide.pladys@ehesp.fr)). Department of Méthode Quantitative en Santé Publique (METIS), EHESP, Avenue du Pr. Leon Bernard, 35043 Rennes, France.

**Supplementary material**

**Table S1. Definition of comorbidities according to ICD-10 codes (In-hospital diagnoses)**

| **Comorbidities** | **ICD-10 codes** |
| --- | --- |
| **Dementia** | F00-F03;F051;G30;G311 |
| **Chronic pulmonary disease** | I278;I279;J40-J47;J60-J67;J684;J701;J703 |
| **Connective tissue disorder** | M05;M06;M315;M32;M33;M34;M351;M353;M360 |
| **Peptic ulcer disease** | K25-K28 |
| **Mild liver disease** | B18;K700-K703;K709;K713K715;K717;K73;K74;K760; K762-K764;K768;K769;Z944 |
| **Moderate or severe liver disease** | I850;I859;I864;I982;K704;K711; K721;K729; K765-K767 |
| **Diabetes without chronic complication** | E100;E101;E106;E108;E109;E110;E111;E116;E118;E119; E120;E121; E126;E128-E131;E136;E138-E141; E146;E148; E149 |
| **Diabetes with chronic complication** | E102-E105;E107;E112-E115;E117; E122-E125;E127;E132-E135;E137; E142-E145;E147 |
| **Hemiplegia or paraplegia** | G041;G114;G801;G802;G81;G82; G830; G831-G834;G839 |
| **Any malignancy** | C00-C26;C30-C34;C37-C41;C43; C45-C58; C60-C76;C81-C85;C88; C90-C97 |
| **Metastatic solid tumor** | C77-C80 |
| **Myocardial infarction** | I21;I22;I252;I255 |
| **Congestive heart failure** | I110;I130;I132;I50 |
| **Peripheral vascular disease** | I70;I71;I731;I738;I739;I771;I790;I792;K551;K558;K559; Z958;Z959 |
| **Cerebrovascular disease** | G45;G46;H340;I60-I69 |

**Table S2. Characteristics at dialysis start of the entire population (n=9052 patients with ESRD) and of the derivation (n=6336) and validation (n=2716) samples.**

|  | **Entire population** | **Derivation** | **Validation sample** |  |
| --- | --- | --- | --- | --- |
|  | **n=9052** | **n=6336** | **n=2716** |  |
|  | **n (%)** | **n (%)** | **n (%)** | **p** |
| **Sex** |  |  |  | 0.495 |
| Men | 5830 (64.4) | 4095 (64.6) | 1735 (63.9) |  |
| Women | 3222 (35.6) | 2241 (35.4) | 981 (36.1) |  |
| **Age (mean ± sd)** | 68.4 (15.08) | 68.4 (15.1) | 68.5 (15.0) | 0.694 |
| **Primary renal disease** |  |  |  | 0.190 |
| Polycystic disease | 463 (5.1) | 329 (5.2) | 134 (4.9) |  |
| Hypertensive and vascular nephropathy | 2433 (26.9) | 1739 (27.4) | 694 (25.6) |  |
| Diabetic nephropathy | 2093 (23.1) | 1459 (23.0) | 634 (23.3) |  |
| Glomerulonephritis | 1106 (12.2) | 794 (12.5) | 312 (11.5) |  |
| Pyelonephritis | 377 (4.2) | 263 (4.2) | 114 (4.2) |  |
| Others | 2580 (28.5) | 1752 (27.7) | 828 (30.5) |  |
| **Albumin (g/dl)** |  |  |  | 0.517 |
| <30 | 1595 (17.6) | 1114 (17.6) | 481 (17.7) |  |
| ≥30 | 6251 (69.1) | 4361 (68.8) | 1890 (69.6) |  |
| *Missing* | 1206 (13.3) | 861 (13.6) | 345 (12.7) |  |
| **Hemoglobin (g/dl)** |  |  |  | 0.796 |
| <10 | 4883 (53.9) | 3412 (53.9) | 1471 (54.2) |  |
| [10-12] | 2638 (29.1) | 1864 (29.4) | 774 (28.5) |  |
| >12 | 1186 (13.1) | 822 (13.0) | 364 (13.4) |  |
| *Missing* | 345 (3.8) | 238 (3.8) | 107 (3.9) |  |
| **BMI (kg/m²)** |  |  |  |  |
| <18.5 | 267 (3.0) | 174 (2.8) | 93 (3.4) |  |
| 18.5-23 | 18.43 (20.4) | 1276 (20.1) | 567 (20.9) |  |
| 23-25 | 1187 (13.0) | 837 (13.2) | 350 (12.9) |  |
| ≥25 | 4221 (46.6) | 2975 (47.0) | 1246 (45.9) |  |
| *Missing* | 1534 (17.0) | 1074 (17.0) | 460 (16.9) |  |
| **Dialysis modality** |  |  |  | 0.768 |
| Hemodialysis | 8213 (90.7) | 5745 (90.7) | 2468 (90.9) |  |
| Peritoneal dialysis | 839 (9.3) | 591 (9.3) | 248 (9.1) |  |
| **Conditions of first dialysis** |  |  |  |  |
| Emergency start | 2769 (30.6) | 1908 (30.1) | 1855 (68.3) |  |
| Planned start | 6283 (69.4) | 4428 (69.9) | 861 (31.7) |  |
| **Smoking status** |  |  |  |  |
| Current/former smoker | 4287 (47.4) | 2999 (47.3) | 1288 (47.4) | 0.140 |
| Never smoker | 3181 (35.1) | 2325 (36.7) | 946 (34.8) |  |
| *Missing* | 1494 (16.5) | 1012 (16.0) | 482 (17.8) |  |
| **Diabetes** |  |  |  | 0.144 |
| Yes | 4140 (45.7) | 2910 (45.9) | 1230 (45.3) |  |
| No | 4872 (53.8) | 3404 (53.7) | 1468 (54.0) |  |
| *Missing* | 40 (0.4) | 22 (0.4) | 18 (0.7) |  |
| **Respiratory insufficiency** |  |  |  | 0.887 |
| Yes | 1678 (18.5) | 1180 (18.6) | 498 (18.3) |  |
| No | 7128 (78.8) | 4989 (78.7) | 2139 (78.8) |  |
| *Missing* | 246 (2.7) | 167 (2.7) | 79 (2.9) |  |
| **Hepatic disease** |  |  |  |  |
| Yes | 233 (2.6) | 6014 (94.9) | 2566 (94.5) |  |
| No | 8580 (94.8) | 164 (2.6) | 69 (2.5) |  |
| *Missing* | 239 (2.6) | 158 (2.5) | 81 (3.0) |  |
| **Cancer** |  |  |  | 0.794 |
| Yes | 1461 (16.1) | 1036 (16.4) | 425 (15.7) |  |
| No | 7382 (81.6) | 5160 (81.4) | 2222 (81.8) |  |
| *Missing* | 209 (2.3) | 140 (2.2) | 69 (2.5) |  |
| **Number of cardiovascular diseases** |  |  |  | 0.843 |
| 0 | 4051 (44.8) | 2829 (44.7) | 1222 (45.0) |  |
| 1 | 2209 (24.4) | 1541 (24.3) | 668 (24.6) |  |
| ≥2 | 2792 (30.8) | 1966 (31.0) | 826 (30.4) |  |
| **Walking disability** |  |  |  | 0.962 |
| Autonomy | 6949 (76.8) | 4862 (76.7) | 2087 (76.8) |  |
| Moderate | 982 (10.9) | 685 (10.8) | 294 (10.9) |  |
| Severe | 388 (4.3) | 270 (4.3) | 118 (4.3) |  |
| *Missing* | 733 (8.1) | 519 (8.2) | 214 (7.9) |  |
| **Physical disabilities** |  |  |  | 0.755 |
| Yes | 1280 (14.1) | 894 (14.1) | 386 (14.2) |  |
| No | 7273 (80.4) | 5085 (80.3) | 2188 (80.6) |  |
| *Missing* | 499 (5.5) | 357 (5.6) | 142 (5.2) |  |

**Table S3. Causes of death after one year of folllow-up (n=1302)**

| **Causes of death** | **n=1302** |
| --- | --- |
|  | **n (%)** |
| **Cardiovascular disease** | 310 (23.8) |
| **Renal disease** | 4 (0.3) |
| **Active malignancy** | 158 (12.1) |
| **Infectious disease** | 199 (15.3 |
| **Cachexia** | 118 (9.1) |
| **Hepatic disease** | 10 (0.8) |
| **Respiratory disease** | 39 (3.0) |
| **Hyperkalemia** | 9 (0.7) |
| **Unknown** | 257 (19.7) |
| **Others** | 198 (15.2) |

**Table S4. Risk factors for 1-year all-cause mortality in the univariate Cox model established in the derivation sample (n=6336), using data from the REIN and SNDS databases.**

|  | **Unadjusted HR**  **(95% CI)** | **p** |
| --- | --- | --- |
| Data from **REIN** | | |
| **Albumin *(vs ≥30g/dl)*** |  |  |
| <30 | 2.24 (1.94-2.58) | <0.001 |
| **Hemoglobin (vs 10-12 g/dl)** |  |  |
| <10 | 1.26 (1.08-1.47) | 0.003 |
| ≥12 | 0.88 (0.69-1.12) | 0.305 |
| **BMI (vs 23-25 kg/m²)** |  |  |
| <18.5 | 1.61 (1.17-2.21) | 0.003 |
| 18.5-23 | 1.05 (0.84-1.30) | 0.664 |
| ≥25 | 0.85 (0.71-1.03) | 0.106 |
| **Tobacco (vs No-smoker)** |  |  |
| Current/Former smoker | 1.01 (0.87-1.17) | 0.944 |
| **Diabetes (vs No)** |  |  |
| Yes | 1.07 (0.94-1.21) | 0.334 |
| **Active malignancies *(vs No)*** |  |  |
| Yes | 2.44 (2.12-2.81) | <0.001 |
| **Hepatic disease (vs No)** |  |  |
| Yes | 1.81 (1.44-2.26) | <0.001 |
| **Respiratory insufficiency (vs No)** |  |  |
| Yes | 1.74 (1.50-2.02) | <0.001 |
| **Walking disability *(vs Autonomy)*** |  |  |
| Moderate | 2.96 (2.51-3.50) | <0.001 |
| Severe | 5.11 (4.21-6.20) | <0.001 |
| **Cardiovascular diseases *(vs 0)^1^*** |  |  |
| 1 | 1.97 (1.65-2.36) | <0.001 |
| ≥2 | 2.86 (2.44-3.35) | <0.001 |
| Data from **SNDS** | | |
| **Diabetes with end-organ damage (vs No)** |  |  |
| Yes | 1.20 (0.98-1.47) | 0.071 |
| **Connective tissue disease (vs No)** |  |  |
| Yes | 1.25 (0.77-2.01) | 0.369 |
| **Ulcer disease (vs No)** |  |  |
| Yes | 1.24 (0.88-1.76) | 0.218 |
| **Mild liver disease** **(vs No)** |  |  |
| Yes | 1.38 (1.01-1.89) | 0.043 |
| **Moderate or severe liver disease (vs No)** |  |  |
| Yes | 2.71 (1.88-3.90) | <0.001 |
| **Any tumor**  **(vs No)** |  |  |
| Yes | 2.13 (1.80-2.52) | <0.001 |
| **Metastatic solid tumor** (vs No) |  |  |
| Yes | 3.08 (2.26-4.20) | <0.001 |
| **Dementia (vs No)** |  |  |
| Yes | 1.72 (1.15-2.58) | 0.009 |
| **Hemiplegia (vs No)** |  |  |
| Yes | 1.32 (0.93-1.85) | 0.117 |

**Figure S1. Calibration curve showing the relationship between the predicted and observed 1-year mortality in the validation sample (Pearson correlation coefficient R²=0.984).**
